# Alveolar Nitric Oxide in Chronic Obstructive Pulmonary Disease—A Two-Year Follow-Up

**DOI:** 10.3390/biomedicines10092212

**Published:** 2022-09-07

**Authors:** Marieann Högman, Andreas Palm, Johanna Sulku, Björn Ställberg, Karin Lisspers, Kristina Bröms, Christer Janson, Andrei Malinovschi

**Affiliations:** 1 Department of Medical Sciences, Respiratory, Allergy and Sleep Research, Uppsala University, 751 85 Uppsala, Sweden; 2 Department of Pharmacy, Uppsala University, 751 23 Uppsala, Sweden; 3 Department of Public Health and Caring Sciences, Family Medicine and Preventive Medicine, Uppsala University, 751 22 Uppsala, Sweden; 4 Department of Medical Sciences, Clinical Physiology, Uppsala University, 751 85 Uppsala, Sweden

**Keywords:** COPD, fraction exhaled nitric oxide and lung function tests, comorbidity, GOLD, mathematical model, gas exchange

## Abstract

Chronic obstructive pulmonary disease (COPD) affects the airways and gas exchange areas. Nitric oxide (NO) production from the airways is presented as F_E_NO_50_ and from the gas exchange areas as alveolar NO (C_A_NO). We aimed to evaluate, over two years, the consistency of the C_A_NO estimations in subjects with COPD. A total of 110 subjects (45 men) who completed the study were included from primary and secondary care settings. C_A_NO was estimated using the two-compartment model. C_A_NO increased slightly during the two-year follow-up (*p* = 0.01), but F_E_NO_50_ remained unchanged (*p* = 0.24). Among the subjects with a low C_A_NO (<1 ppb) at inclusion, only 2% remained at a low level. For those at a high level (>2 ppb), 29% remained so. The modified Medical Research Council dyspnoea scale (mMRC) score increased at least one point in 29% of the subjects, and those subjects also increased in C_A_NO from 0.9 (0.5, 2.1) ppb to 1.8 (1.1, 2.3) ppb, *p* = 0.015. We conclude that alveolar NO increased slightly over two years, together with a small decline in lung function. The increase in C_A_NO was found especially in those whose levels of dyspnoea increased over time.

## 1. Introduction

Chronic obstructive pulmonary disease (COPD) affects the conducting airways and gas exchange areas. Post-bronchodilator spirometry sets a COPD diagnosis in individuals with typical respiratory symptoms such as dyspnoea and chronic cough, often together with a history of relevant exposure to smoke, usually cigarette smoke. Emphysema with the destruction of lung parenchyma can develop after many years of smoking and cannot be reversed.

In a meta-analysis, the fraction of exhaled nitric oxide (F_E_NO) was slightly increased in persons with COPD compared to healthy controls [1]. F_E_NO is further increased if there is an eosinophilic inflammation, such as type-2 inflammation [2]. Tobacco smoke will interfere with nitric oxide (NO) production in the airway epithelia. Patients with COPD who are currently smoking will therefore have a lower F_E_NO than ex-smokers. Additionally, ex-smokers have lower values than never-smokers [3]. Thus, the clinical significance of F_E_NO in stable COPD patients is unclear, as was summarised in a recent scoping review [4].

The NO production in the lungs can be traced to the exhalation gas. Fractional exhaled nitric oxide at 50 mL/s (F_E_NO_50_) represents the NO production in the airways, and the alveolar NO (C_A_NO) represents production from the gas exchange areas. In COPD, there is an involvement in the lung parenchyma, which can manifest as emphysema. Higher values of C_A_NO in COPD patients have been reported [5,6,7,8], but also values that did not differ from healthy controls [9]. There are also methodological issues associated with the estimation of C_A_NO, which were reviewed in 2017 by a European Respiratory Society task force [10]. The task force recommended two methods to determine estimation: the linear method by Tsoukias & George and the non-linear Högman-Meriläinen algorithm. Both methods refer to Fick’s first law of diffusion, where a bolus of gas (alveolar gas) transported up into the conducting airways picks up NO driven by a concentration gradient from the bronchial wall. These methods are explained, and the equations for the calculations can be found together with the usefulness of these methods in respiratory diseases in Högman et al., 2014, 2017 [11,12].

A limited number of studies have followed C_A_NO values in patients with COPD over a more extended period. Lehouck et al. followed 22 patients in stable condition for four months. There were no statistically significant differences in the C_A_NO values compared to those at inclusion [9]. Lázár et al. investigated patients in a stable disease state and patients experiencing an acute exacerbation, and they found that the C_A_NO values were elevated compared to healthy controls [13]. However, no difference was found between the stable and the exacerbated patients. In 26 patients, there was no difference between the C_A_NO at the time of the acute exacerbation and discharge from the hospital. Two other studies looked at the effect of corticosteroid treatment after one week [14] and after four weeks [15]. Both studies showed no difference in C_A_NO between the visits.

This study aimed to evaluate, over two years, the consistency of the C_A_NO estimations in subjects with COPD and the associations of the C_A_NO changes to the clinical progression of the disease.

## 2. Materials and Method

### 2.1. Study Design and Subjects

**Figure 1:**
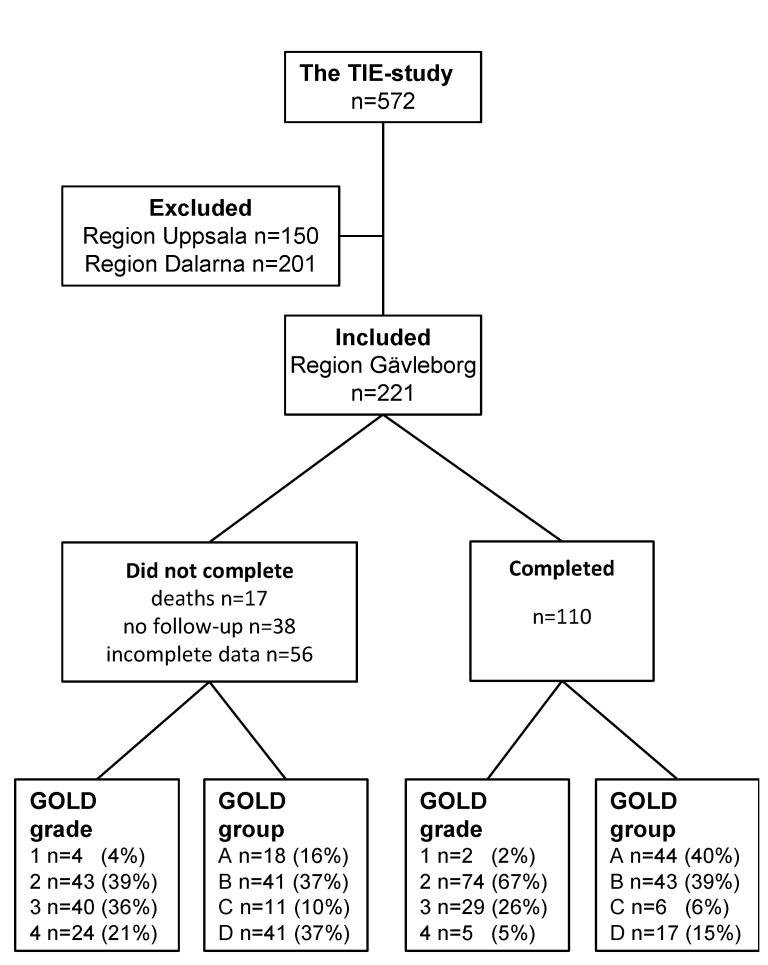
Flow chart of excluded and included COPD subjects. Subjects were grouped according to whether they completed or did not complete the study. Further grouping at inclusion was according to the GOLD 2021 assessment of airflow limitations (GOLD grade) and symptom/risk of exacerbation (GOLD group) (https://goldcopd.org, accessed on 1 March 2022).

The research subjects were recruited from the Swedish multicentre study: Tools Identifying Exacerbations in COPD (TIE-study) [16]. Those included were participants over the age of 40 with a diagnosis of COPD from primary and secondary care settings who came from one of the research centres that could measure C_A_NO at inclusion and at the one and two-year follow-ups (Figure 1). The measurements were performed only when the study participants were in a stable disease state, i.e., no exacerbation within the last three weeks. Recruitment to the study occurred from September 2014 until October 2016. The study was completed in October 2018.

### 2.2. Methods

The COPD diagnosis was set by a physician and confirmed by spirometry. The measurement was obtained using a post-bronchodilator (400 µg salbutamol) forced expiratory volume in one second (FEV_1_) divided by the highest value of vital capacity (VC) or forced vital capacity (FVC) with a ratio of <0.70 (SpiroPerfect spirometer, Welch Allyn, Skaneateles Falls, NY, USA). FEV_1_ and FVC are presented as per cent predicted using Swedish reference values [17,18].

According to the 2005 standardised measurements recommendation, the measurement of exhaled NO was performed at a flow of 50 mL/s (F_E_NO_50_) [19]. In addition to F_E_NO_50_, exhaled NO at flows of 20, 100, and 300 mL/s were measured in duplicate for the non-linear Högman-Meriläinen algorithm (HMA) modelling of NO exchange. The NO analyser was equipped with the software for the HMA estimation (Eco Medics CLD 88, Eco Medics, Dürnten, Switzerland). HMA estimates the C_A_NO, airway wall NO content (C_aw_NO), the diffusing capacity of NO from the airway wall (D_aw_NO), and F_E_NO_50_. For quality control, the measured and the estimated F_E_NO_50_ were compared for a difference not exceeding 5 ppb [10]. For subjects who could not perform the HMA, the linear modelling method was applied with flows of 100, 200, and 300 mL/s and an r-value > 0.95 [10].

Blood cell counts analysing neutrophils (B-Neu) and eosinophils (B-Eos) were performed (Cell-Dyn 4000, Abbott, Abbott Park, IL, USA and Sysmex XN-10, Sysmex America Inc, Lincoinshire, IL, USA). Additionally, questionnaires including the COPD Assessment Test (CAT), the Clinical COPD Questionnaire (CCQ) and the modified Medical Research Council Dyspnoea Scale (mMRC) were used [20]. A clinical difference in CAT was set at ≥2 points [21] and CCQ at ≥0.4 points [22]. For statistical comparisons, mMRC scores were grouped as <2 and ≥2. Questions regarding demographics, smoking habits, comorbidities and inhaled COPD treatment were also assessed. The research nurse reviewed the answers to the questionnaires with the research subjects to assure accuracy.

### 2.3. Data Analysis

To classify the COPD subjects’ disease severity, the GOLD risk assessment version 2021 with assessments of airflow limitations and symptoms/risk of exacerbations (CAT scale) was used together with the history of exacerbations/hospitalisations. An exacerbation was defined as an unscheduled health care visit, and/or a course of oral corticosteroids, and/or a course of antibiotics due to COPD deterioration (questionnaire assessed). Information about hospitalisation admittance was retrieved from hospital records. A questionnaire gathered the exacerbation history for the year prior to each visit.

Binominal test, Pearson χ^2^-test, Kolmogorov–Smirnov normality test, and non-parametric tests, i.e., McNemar χ^2^-test, Mann–Whitney U test, Friedman’s test and Spearman’s rho (SPSS, v. 24 for Windows, SPSS Inc., Chicago, IL, USA) were used for the statistical calculations. Descriptive statistics are given as frequencies and percentages, mean ± SD, or median with lower quartile (Q1) and upper quartile (Q3). A *p*-value of *p* < 0.05 was considered significant.

## 3. Results

Of the 221 included subjects, 111 did not complete the study. Non-completion was due to death, lack of participation in all follow-up visits, or incomplete data collection (Figure 1). F_E_NO_50_ could not be collected from six subjects, and the C_A_NO estimations were missing from 51 subjects. More severe disease was found in the subjects who did not complete the study, as validated by lung function, higher B-Neu levels, lower F_E_NO_50_ and higher C_A_NO values. The symptom burden according to the CAT, CCQ, and mMRC was also higher, and they had a greater number of comorbidities, exacerbations, and treatments with triple therapy (an inhaled corticosteroid in combination with a long-acting beta-2-agonist and a long-acting muscarinic antagonist) than the subjects who completed the study, see Table 1. The C_A_NO and F_E_NO_50_ were 2.3 (0.6, 3.5) and 12 (5, 15) ppb, respectively in participants who died before completing the study. The 51 subjects whose NO modelling was missing had a FEV_1.0_ of 1.33 (0.94, 1.56) L and a FVC of 2.40 (1.97, 2.96) L.

A total of 110 subjects completed the two-year follow-up. They were aged 68 ± 8 years and 59% were women. Among the comorbidities reported were: asthma (33%) and chronic bronchitis (25%). However, the distribution of C_A_NO was not different from those subjects who did not report asthma or chronic bronchitis, *p* = 0.81 and *p* = 0.26, respectively. Other comorbidities were hypertension (46%), anxiety/depression (21%), heart disease (19%), and diabetes (5%). Additional characteristics of the study participants are presented in Table 2 and Figure 1.

Most of our subjects could perform the multiple flows for the HMA to estimate the C_A_NO, but the linear model was used for two subjects at the inclusion, for seven subjects at the one-year visit and for 24 subjects at the two-year visit. There was no statistically significant change in F_E_NO_50_ between the visits. F_E_NO_50_ was lower in subjects who were currently smoking at inclusion compared to ex-smokers, F_E_NO_50_ 9 (6, 16) ppb and 15 (11, 24) ppb respectively, *p* = 0.004. C_aw_NO was also lower, 34 (21, 94) ppb and 74 (36, 154) ppb respectively, *p* = 0.018, while D_aw_NO and C_A_NO were not affected by smoking status (*p* = 0.56 respectively *p* = 0.27). C_A_NO increased during the study period while the other parameters of the HMA did not change (Table 2). C_A_NO had no correlation to age. There was no statistically significant difference in C_A_NO between females and males, 1.3 (0.7, 2.3) ppb and 1.3 (0.5, 2.0) ppb respectively, *p* = 0.38. C_A_NO was 1.3 (0.6, 2.1) ppb in subjects without ICS and 1.3 (0.5, 2.2) ppb in subjects taking ICS, *p* = 0.68. During the two-year follow-up, the FEV_1_ % predicted was not significantly changed, but the FVC % predicted decreased slightly (*p* < 0.001), see Table 2. There were no correlations between lung function and F_E_NO_50_ or C_A_NO at any point in time. Additionally, there was no correlation between the change in F_E_NO_50_ or C_A_NO and the change in lung function.

**Figure 2:**
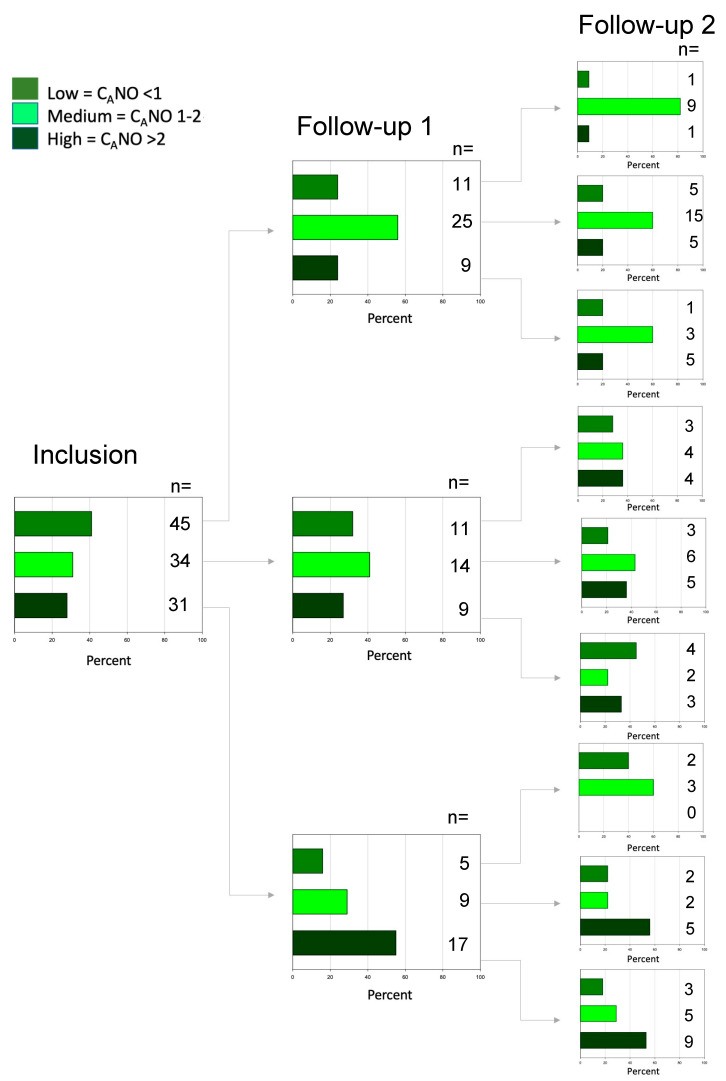
The estimation of C_A_NO at inclusion and each follow-up during the two-year period. The subjects are grouped in low <1 ppb, medium 1–2 ppb, and high >2 ppb.

To evaluate the consistency of the C_A_NO measurements, we divided the participants into three groups: low <1 ppb, medium 1–2 ppb, and high >2 ppb (Figure 2). Only 2% of the subjects who had a low C_A_NO at inclusion consistently remained at a low level at the last follow-up. In the medium group 18% remained at the same level, and in the high C_A_NO group 29% remained the same.

**Figure 3:**
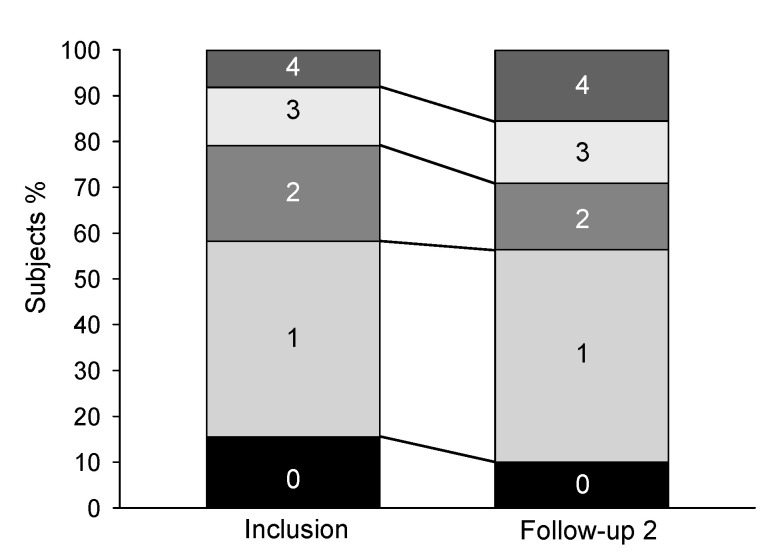
The modified Medical Research Council dyspnoea scale (mMRC) (ordinal scale 0–4) at inclusion and after two years.

**Figure 4:**
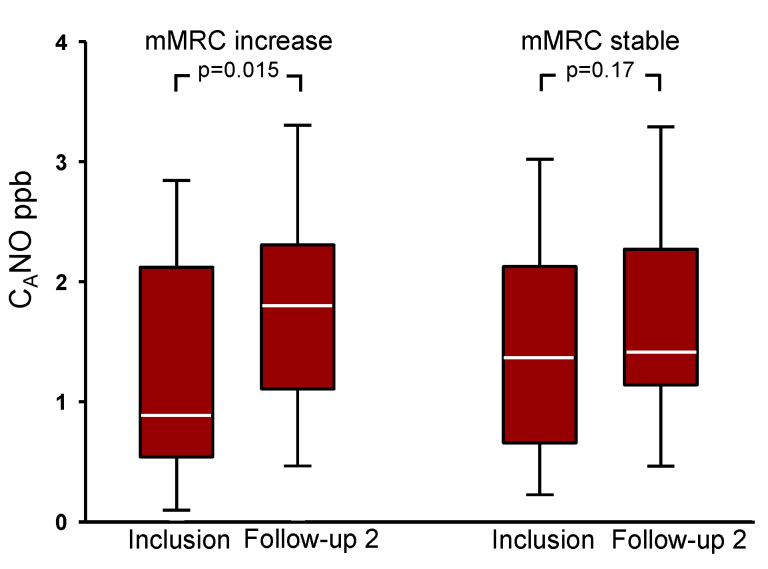
C_A_NO values for the subjects that increased their modified Medical Research Council dyspnoea scale (mMRC) by 1–3 points (n = 32, left) and the subjects that had a stable mMRC, minus 1 or the same value (n = 78, right) at inclusion and after two years. The horizontal line in each box corresponds to the median value, the upper and lower margins correspond to Q1 and Q3, and the whiskers correspond to the 10th and 90th percentiles.

The symptom burden assessed by CAT and CCQ did not change during the two-year follow-up period (Table 2). A clinical increase in CAT scores was seen in 44 subjects, but there was no difference in the change in C_A_NO in those with and those without an increase (*p* = 0.38). In CCQ scores, a clinical decrease was seen in 36 subjects also without a difference in the change in C_A_NO (*p* = 0.08). There were changes in the mMRC dyspnoea scores over the study period, with a shift toward higher values as illustrated in Figure 3 and seen in Table 2. The subjects were divided into two groups, one that increased their mMRC scores by 1–3 points (n = 32) and another that scored minus 1 or had the same value (n = 78) between inclusion and the two-year follow-up. The distribution of C_A_NO at inclusion was the same for the two mMRC groups. As seen in Figure 4, the subjects who had an increase in their mMRC, also had an increase in C_A_NO.

The inhaled COPD treatment of our participants that completed the study was similar during the study period (Table 2). The frequency of exacerbations for the subjects with ≥1 exacerbation(s) the year prior to inclusion decreased at the one and two-year follow-ups (Table 2).

## 4. Discussion

This study showed that over a period of two years, subjects with COPD who had stable treatment had a progression of the disease; as evidenced by slightly lower FVC measurements, higher C_A_NO values, and a shift towards higher dyspnoea scores. The subjects who increased their dyspnoea scores had a higher C_A_NO. Only a third of our subjects with a higher C_A_NO value, defined as above 2 ppb, remained at the same level after two years.

When C_A_NO values from this study were compared to those from healthy subjects, the values from the COPD subjects were found to be slightly lower. The subjects completing the study aged 47–83 years had a C_A_NO of 1.3 that increased to 1.7 ppb, the subjects not completing aged 40–87 years had at inclusion a C_A_NO of 1.5 ppb, and the healthy comparison subjects from the literature aged 50–78 years had 2.2 (1.5, 2.9) ppb [11]. C_A_NO increases with age in healthy subjects [11], but there was no correlation between age and C_A_NO among our COPD subjects.

C_A_NO values have been found to be not increased in COPD patients [9], but contradictory results with higher values have also been found [5,6,23]. These divergent results might be related to differences in the populations studied, but also to methodological aspects. For example, the lowest flow to use with the linear method is 100 mL/s [10], and using a flow lower than that will give a falsely high C_A_NO level. However, we found higher C_A_NO values in a small sample of COPD patients when using the HMA as in the present study [8]. This difference is, therefore, most likely due to the subjects being in different stages of the disease, since C_A_NO increased with disease progression. The subjects who did not complete the study and had a higher disease burden had higher C_A_NO values than the subjects who completed the study.

Good consistency of C_A_NO estimation over the follow-up period was lacking. Only 29% of our subjects with a higher C_A_NO value, defined as above 2 ppb, remained at the same level after two years. However, in this study, there was an increase in C_A_NO over time. The increase was mainly seen in participants that had increased problems with dyspnoea. In COPD, the alveolar region is involved with the formation of emphysema, which is known to affect the gas exchange and ventilation to perfusion ratio (VA/Q) matching [24], which starts already in GOLD stage 1 (FEV_1_-% predicted ≥80) [25]. Further investigation is needed to determine if the loss of lung volume causes a compensatory upregulation of NO in the peripheral lung. McCurdy et al. studied physical performance and C_A_NO in COPD patients. They found higher C_A_NO values with shorter travel distances in a walking test [7], which gives the hypothesis for compensatory upregulation credibility. Another possibility is that the uptake of the inhaled NO that is produced in the airways is not homogenous, such that some of the inhaled NO is exhaled at high flows giving a higher C_A_NO.

In contrast to the NO from the airways, as well as the content of NO in the airway wall, the effect of smoking was not seen in C_A_NO or in the diffusing capacity of NO from the airway wall in this study. That C_A_NO is not affected by smoking has been shown previously [3]. The contrary has also been shown, but then the highest flow was too low, which allowed NO to be picked up from the airway [9,12]. In a study examining smoking cessation over a four-week period, the airway NO gradually increased, while C_A_NO remained unchanged [26]. This suggests that C_A_NO might be a useful marker since many COPD patients are still smoking or are trying to quit.

Most of our research subjects could perform the NO modelling with multiple flows. There were only two subjects in the completed group at inclusion that could not perform the lowest flow of 20 mL/s. It was more difficult at the two-year follow-up since, for 24 subjects, we had to use the linear model that excludes the low flow. Among all of the subjects, both completed and non-completed, NO modelling was missed in 22% for whatever reason (performance, analyser, or simply not done). Lazar et al. missed 7% in their stable subjects due to an inability to complete a flow of 250 mL/s and as much as 32% in the subjects that had an exacerbation at inclusion [13]. Karvonen et al. had a higher rate of successful results with the linear model compared to the HMA [27]. We can conclude that NO modelling with multiple flows cannot be accomplished in all patients.

A limitation of this study was that we were not able to follow the subjects who did not complete the study and had more severe disease. We do not know what the reasons were for the missing values, but during the follow-up period, there were more subjects that required the use of higher flows for the NO analysis. This points to a more severe disease as the possible reason. It could be seen as a limitation that different methods of estimating C_A_NO were used, but they are partially based on the same measurements, and it has been found that the estimation of C_A_NO with the linear and the non-linear methods do not differ [12]. To strengthen the clinical value of C_A_NO, it would have been interesting to evaluate both hyperinflation and lung diffusion capacity (DLCO) from a pulmonary function perspective, and have a computerized tomography assessment of emphysema in the present study.

The strength of this study is that we have longitudinal data on estimated C_A_NO over a two-year period and could analyse changes over time in relation to changes in disease burden. Another strength is that the subjects were examined in a stable disease state at all time points. Our study population involved patients from both primary and secondary care settings, making these results relevant to most of the patients. We have used validated questionnaires at all three visits and the same research nurse reviewed these questionnaires with the subjects. Finally, COPD was physician-diagnosed and verified by spirometry at the inclusion in the study.

## 5. Conclusions

Alveolar NO increased slightly over the follow-up period, together with a small decline in lung function. There was a lack of consistency in the alveolar NO values, with only a third of our subjects with higher C_A_NO values, remaining at the same level after two years. The increase in C_A_NO was found especially in those whose levels of dyspnoea increased over time.

## Figures and Tables

**Table 1:** Characteristics of the study subjects at inclusion who completed or did not complete the study.

	Did Not Complete n = 111	Completed n = 110	*p*-Value
Women n (%)	67 (60%)	65 (59%)	0.847
Age years	69 ± 8	68 ± 8	0.113
Current daily smokers n (%)	25 (23%)	28 (26%)	0.506
BMI	26 (23, 30)	27 (23, 32)	0.185
*Comorbidity*			
Asthma	45%	33%	0.060
Chronic bronchitis	45%	25%	**0.002**
Heart infarction/angina	11%	12%	0.813
Heart failure	6%	1%	**0.032**
Heart fibrillation	19%	6%	**0.005**
Hypertension	47%	46%	0.836
Diabetes	13%	5%	**0.034**
Anxiety/depression	30%	21%	0.132
*Lung function*			
FEV_1.0_ L	1.17 (0.79, 1.51)	1.53 (1.24, 1.86)	**<0.001**
FEV_1.0_ % predicted	45 (31, 58)	57 (49, 67)	**<0.001**
FVC L	2.38 (1.89, 3.16)	2.83 (2.47, 3.45)	**<0.001**
FVC % predicted	57 (47, 74)	67 (61, 76)	**<0.001**
*Inflammatory markers*			
B-Neu 10^9^/L	5.4 (4.3, 6.1)	4.3 (3.5, 4.9)	**<0.001**
B-Eos 10^9^/L	0.14 (0.08, 0.23)	0.18 (0.10, 0.28)	0.094
*Exhaled NO*			
F_E_NO_50_ ppb	11 (6, 17) ^1^	14 (9, 21)	**0.006**
C_A_NO ppb	1.5 (0.9, 2.7) ^2^	1.3 (0.6, 2.1)	**0.043**
C_aw_NO ppb	35 (16, 95) ^2^	65 (30, 136)	**0.007**
D_aw_NO mL/s	22 (10, 34) ^2^	15 (7, 30)	0.084
*Symptom burden*			
CAT	14 (9, 22)	11 (6, 16)	**<0.001**
mMRC ≥ 2	65 (59%)	46 (42%)	**0.013**
CCQ	2.0 (1.1, 3.1)	1.3 (0.7, 2.1)	**<0.001**
*Exacerbations*			
Questionnaire ≥ 1 (%)	67 (61%)	48 (44%)	**<0.001**
*Inhaled treatment*			
ICS + LABA + LAMA ^3^	73 (66%)	46 (42%)	**<0.001**

^1^ Missing 6 subjects, ^2^ missing 51 subjects; ^3^ regular treatment with inhaled corticosteroids (ICS) in combination with long-acting beta-2-agonist (LABA), and long-acting muscarinic antagonist (LAMA). Body mass index (BMI), forced expiratory volume at 1 s (FEV_1.0_), forced vital capacity (FVC), blood neutrophils (B-Neu), blood eosinophils (B-Eos), fraction of exhaled nitric oxide at 50 mL/s (F_E_NO_50_), alveolar NO (C_A_NO), airway wall NO content (C_aw_NO), diffusing capacity of NO from the airway wall (D_aw_NO), COPD Assessment Test (CAT), modified Medical Research Council dyspnoea scale (mMRC), Clinical COPD Questionnaire (CCQ). Data are given in percentage, median (Q1, Q3), or mean ± SD.

**Table 2:** Characteristics of the subjects completing the study at inclusion, and their one and two-year follow-ups.

	Inclusion n = 110	1-Year n = 110	2-Year n = 110	*p*-Value
Current daily smokers n (%)	28 (26%)	22 (20%)	27 (24%)	1.0
BMI	27 (23, 32)	27 (23, 31)	27 (24, 31)	0.559
*Lung function*				
FEV_1.0_ L	1.53 (1.24, 1.86)	1.53 (1.20, 1.94)	1.49 (1.15, 1.89)	**0.024**
FEV_1.0_ % predicted	57 (49, 67)	58 (48, 68)	56 (46, 66)	0.429
FVC L	2.83 (2.47, 3.45)	2.78 (2.27, 3.18)	2.68 (2.23, 3.27)	**<0.001**
FVC % predicted	67 (61, 76)	66 (57, 76)	65 (56, 76)	**0.007**
*Inflammatory markers*				
B-Neu 10^9^/L	4.3 (3.5, 4.9)	4.3 (3.2, 5.3)	4.2 (3.6, 5.4)	0.890
B-Eos 10^9^/L	0.18 (0.10, 0.28)	0.17 (0.11, 0.29)	0.16 (0.11, 0.24)	0.072
*Exhaled NO*				
F_E_NO_50_ ppb	14 (9, 21)	14 (9, 23)	13 (8, 19)	0.238
C_A_NO ppb	1.3 (0.6, 2.1)	1.5 (1.0, 2.2)	1.7 (1.1, 2.3)	**0.013**
C_aw_NO ppb	65 (30, 136)	49 (21, 111)	52 (25, 96)	0.089
D^aw^NO mL/s	15 (7, 30)	18 (9, 36)	16 (6, 33)	0.553
*Symptom burden*				
CAT	11 (6, 16)	10 (6, 15)	11 (7, 17)	0.523
mMRC n 0/1/2/3/4	17/47/23/14/9	16/48/20/14/12	11/51/16/15/17	
CCQ	1.3 (0.7, 2.1)	1.3 (0.8, 2.1)	1.3 (0.8, 2.4)	0.537
Exacerbations ≥ 1/year	48 (44%)	25 (23%)	29 (27%)	**0.002**
*Treatment*				
No regular treatment n (%)	21 (19%)	22 (20%)	18 (16%)	0.863
Bronchodilators ^1^ n (%)	26 (24%)	22 (20%)	27 (25%)	
ICS ^2^ n (%)	63 (57%)	63 (57%)	64 (58%)	
SABA last week	50 (45%)	48 (44%)	47 (43%)	0.584

^1^ Regular treatment with long-acting beta-2-agonist and/or long-acting or short-acting muscarinic antagonist alone or in combination, ^2^ regular treatment with inhaled corticosteroid alone or in any combination with bronchodilators. Body mass index (BMI), forced expiratory volume at 1 s (FEV_1.0_), forced vital capacity (FVC), blood neutrophils (B-Neu), blood eosinophils (B-Eos), fraction of exhaled nitric oxide at 50 mL/s (F_E_NO_50_), alveolar NO (C_A_NO), airway wall NO content (C_aw_NO), diffusing capacity of NO from the airway wall (D_aw_NO), COPD Assessment Test (CAT), modified Medical Research Council dyspnoea scale (mMRC), Clinical COPD Questionnaire (CCQ), inhaled corticosteroids (ICS), short-acting beta-2-agonist (SABA). Data are given in percentage or median (Q1, Q3).

## Data Availability

Data cannot be made freely available as they are subject to secrecy in accordance with the Swedish Public Access to Information and Secrecy Act, but can be made available to researchers upon request (subject to a secrecy review).

## Abbreviations

B-Eos	blood eosinophils
B-Neu	blood neutrophils
BMI	body mass index
C_A_NO	alveolar NO
CAT	COPD Assessments Test-scores
C_aw_NO	NO content in airway wall
COPD	chronic obstructive pulmonary disease
D_aw_NO	NO diffusion capacity over airway wall
F_E_NO_50_	fraction of exhaled NO at 50 mL/s
FEV_1_	forced expiratory volume in 1 s
FVC	forced vital capacity
HMA	Högman Meriläinen algorithm
mMRC	modified Medical Research Council dyspnoea scale
NO	nitric oxide
